# Redox-sensitive MAPK and Notch3 regulate fibroblast differentiation and activation: a dual role of ERK1/2

**DOI:** 10.18632/oncotarget.9667

**Published:** 2016-05-27

**Authors:** Jun-Mei Lai, Xiong Zhang, Fang-Fang Liu, Rui Yang, Shen-Yu Li, Lan-Bing Zhu, Ming Zou, Wen-Hsing Cheng, Jian-Hong Zhu

**Affiliations:** ^1^ Department of Preventive Medicine, Wenzhou Medical University, Wenzhou, Zhejiang 325035, China; ^2^ Department of Geriatrics and Neurology, the Second Affiliated Hospital, Wenzhou Medical University, Wenzhou, Zhejiang 325035, China; ^3^ Key Laboratory of Watershed Science and Health of Zhejiang Province, Wenzhou Medical University, Wenzhou, Zhejiang 325035, China; ^4^ Department of Food Science, Nutrition and Health Promotion, Mississippi State University, Mississippi State, MS, 39762, USA; ^5^ Department of Rehabilitation Medicine, Zhejiang Provincial People's Hospital, Hangzhou, Zhejiang 310014, China

**Keywords:** differentiation, ROS, MAPK, Notch3, lung fibrosis

## Abstract

Myofibroblastic transformation, characterized by upregulation of α-smooth muscle actin in response to profibrotic agents such as TGF-β1, is considered as a major event leading to fibrosis. The mechanistic basis linking myofibroblast differentiation to idiopathic pulmonary fibrosis and the disease treatment remain elusive. In this study, we studied roles of MAPK, Notch, and reactive oxygen species (ROS) during the differentiation of IMR-90 lung fibroblasts at basal level and induced by TGF-β1. Our results demonstrated that ROS-dependent activation of p38, JNK1/2 and Notch3 promoted basal and TGF-β1-induced differentiation and expression of extracellular matrix proteins. In stark contrast, ERK1/2 was suppressed by ROS and exhibited an inhibitory effect on the differentiation but showed a weak promotion on the expression of extracellular matrix proteins. TGF-β1-induced Notch3 expression depended on p38 and JNK1/2. Interestingly, Notch3 was also downstream of ERK1/2, suggesting a complex role of ERK1/2 in lung function. Our results suggest a novel ROS-mediated shift of dominance from the inhibitory ERK1/2 to the stimulatory p38, JNK1/2 and Notch3 during the pathological progression of IPF. Thus, targeting ERK1/2 signaling for activation and p38, JNK1/2 and Notch3 for inhibition may be of clinical potential against lung fibrosis.

## INTRODUCTION

Idiopathic pulmonary fibrosis (IPF), a progressive and irreversible respiratory disease of unknown cause [1], is characterized by failure of alveolar re-epithelialization, persistence of myofibroblasts, deposition of extracellular matrix, and destruction of lung architecture [2]. Epithelial cells in IPF patients undergo chronic inflammation and release cellular mediators and cytokines, which in turn induce fibroblast migration, proliferation, and differentiation towards myofibroblasts [3,4]. Accumulation and persistence of myofibroblasts play central roles in lung fibrosis through pathophysiological events including the formation of fibroblast foci, excessive matrix deposition, and decreased compliance of lung parenchyma [5,6].

Transforming growth factor β1 (TGF-β1), a potent cytokine, mediates signaling events during the pathogenesis of pulmonary fibrosis by the stimulation of fibroblast differentiation, extracellular matrix deposition, and tensile force in myofibroblasts [1,7,8]. Through interactions with Notch signaling, reactive oxygen species (ROS) and mitogen-activated protein kinases (MAPKs) including ERK, p38 and JNK, TGF-β1 signaling regulates various cellular processes such as vascular smooth muscle differentiation and epithelial-mesenchymal transition [9–11]. It is becoming clear that the differentiation towards myofibroblasts is a key pathophysiological event leading to IPF [12]. Although activation of TGF-β1 is known to be critical for differentiation into myofibroblasts [13], the underlying molecular mechanisms remain elusive. Herein, we have comprehensively investigated roles of ROS, MAPKs and Notch at basal and TGF-β1-ativated conditions during the differentiation processes.

## RESULTS

### p38, JNK and ERK are upstream of Notch3 signaling upon TGF-β1-induced differentiation of IMR-90 fibroblasts

**Figure 1:**
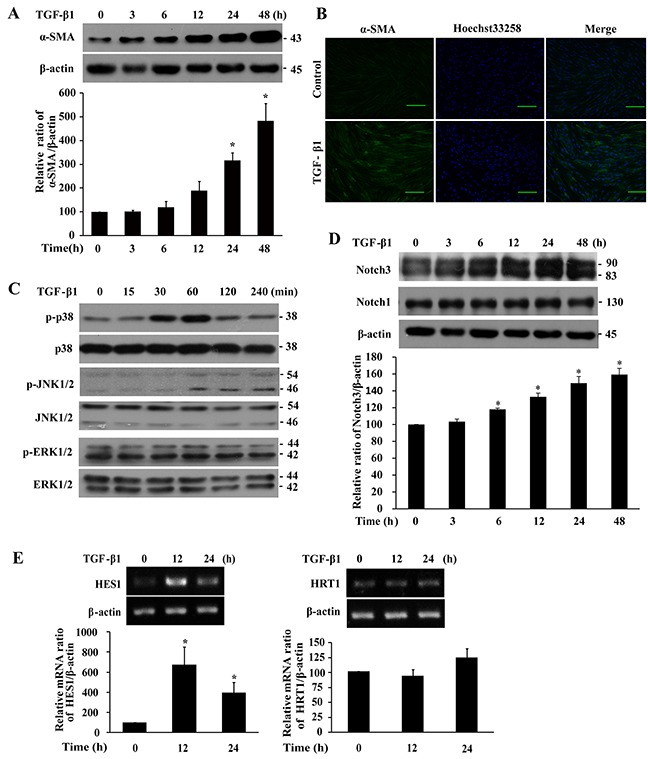
TGF-β1 induces differentiation, p38 and JNK1/2 phosphorylation, and Notch3 expression in IMR-90 fibroblasts Cells were serum-starved overnight prior to treatment with TGF-β1 (200 pM) for the indicated duration. **A.** Western analysis of α-SMA levels. **B.** Immunofluorescent analysis of α-SMA expression. Green, α-SMA; blue, nuclei; bar size, 100 μm. **C.** Western analysis of p38, JNK1/2 and ERK1/2 phosphorylation. **D.** Western analysis of Notch3 and Notch1 levels. **E.** RT-PCR analysis of *HES1* and *HRT1* mRNA levels. The bands were quantified, normalized by β-actin or the respective unphosphorylated protein, and presented relative to 0 h (100%) as means ± SEM (n = 3-4). *, *P* < 0.05 vs 0 h.

**Figure 2:**
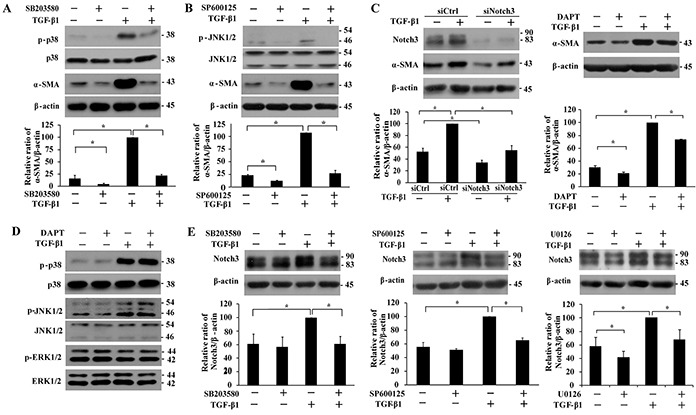
Analyses of the effect of p38, JNK1/2 and Notch3 inhibitions on TGF-β1-induced α-SMA expression and the regulation between MAPKs and Notch3 signaling in IMR-90 fibroblasts Cells were serum-starved overnight prior to treatment with TGF-β1 (200 pM). **A-B.** Effect of pretreatment with p38 inhibitor SB203580 (10 μM, 1 h) and JNK inhibitor SP600125 (20 μM, 1 h) on p38 and JNK1/2 phosphorylation and α-SMA level after TGF-β1 treatment. **C.** Impact of Notch3 siRNA knockdown or inhibitor DAPT (10 μM, 1 h) on TGF-β1-induced α-SMA expression. Cells were transfected with a scrambled control (siCtrl) or Notch3 siRNA (siNotch3) for 24 h prior to treatment with TGF-β1 for 48 h. **D.** Effect of DAPT pretreatment (10 μM, 1 h) on TGF-β1 (1 h)-induced p38 and JNK1/2 activation, as well as ERK1/2 activity. **E.** Effect of p38 inhibitor SB203580 (10 μM, 1 h), JNK inhibitor SP600125 (20 μM, 1 h) and ERK inhibitor U0126 (10 μM, 1 h) on Notch3 expression after TGF-β1 treatment for 48 h. The protein levels of α-SMA and Notch3 were quantified, normalized by β-actin and presented relative to the one treated with TGF-β1 alone (100%) as means ± SEM (n = 3-4). *, *P* < 0.05.

To build a cellular model of fibroblast differentiation mimicking pulmonary fibrosis, IMR-90 fibroblasts were treated with TGF-β1 (200 pM) and followed by a 48-h time course. Results from Western (Figure 1A) and immunofluorescent (Figure 1B) analyses showed time-dependent induction of alpha-smooth muscle actin (α-SMA), a marker of differentiated myofibroblasts, upon TGF-β1 treatment. Analyses of MAPK and Notch activation in TGF-β1-treated cells demonstrated time-dependent phosphorylation of p38 and JNK1/2, but not ERK1/2 (Figure 1C), and elevated expression of Notch3 but not Notch1 protein levels (Figure 1D). Interestingly, we observed two Notch3 bands (90 and 83 kDa, Figure 1D) whose relative abundance varied slightly with passage of cells (data not shown). They were authentic Notch3 proteins because Notch3 siRNA knockdown effectively down-regulated the expression of both bands (Figure 2C). Analyses of Notch3 downstream targets showed an up to 6-fold increase in *HES1* but not *HRT1* mRNA levels upon TGF-β1 treatment (Figure 1E), suggesting that HES1 was more responsive than HRT1 to Notch3 signal in the fibroblasts.

TGF-β1-induced phosphorylation of p38 and JNK1/2 were blocked by pre-treatment with SB203580 and SP600125, respectively, thus validating efficacy of the kinase inhibitors. Pre-treatment with SB203580 or SP600125 reversed TGF-β1-induced expression of α-SMA (Figure 2A and 2B). Notch3 siRNA knockdown or treatment with a Notch inhibitor DAPT suppressed TGF-β1-induced expression of α-SMA protein, but the former is more effective than the latter (Figure 2C). Disrupted p38, JNK1/2 and Notch3 signaling also suppressed α-SMA protein expression at basal levels (Figure 2A-2C). We further determined the signaling cascade between p38/JNK and Notch3. Pre-treatment with DAPT did not affect TGF-β1-induced phosphorylation of p38, JNK1/2 or ERK1/2 (Figure 2D). On the other hand, pre-treatment of SB203580 or SP600125 repressed TGF-β1-induced up-regulation of Notch3 protein expression (Figure 2E, left and middle panels), indicating that p38 and JNK resided upstream of Notch3 signaling in the regulation of fibroblast differentiation. Although ERK1/2 was not activated by TGF-β1 (Figure 1C), pre-treatment with U0126 suppressed Notch3 expression in the absence and presence of TGF-β1 (Figure 2E, right panel), suggesting that ERK was upstream of Notch3 independent of TGF-β1. Altogether, p38, JNK and ERK act upstream of Notch3 signaling in TGF-β1-induced fibroblast differentiation, but ERK is additionally positioned upstream of Notch3 in the absence of TGF-β1 induction.

### TGF-β1-induced fibroblast differentiation and activation of p38, JNK1/2 and Notch3 are redox-dependent

**Figure 3:**
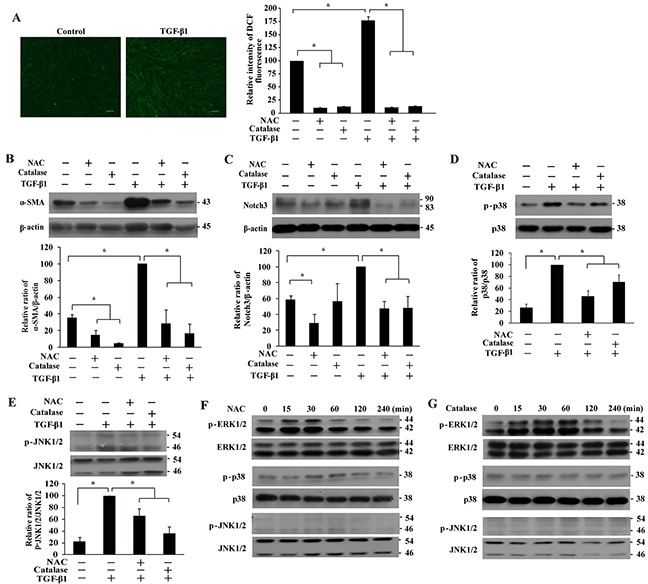
Effect of NAC and catalase pretreatment on basal and TGF-β1-induced α-SMA, MAPKs and Notch3 expression in IMR-90 fibroblasts **A.** Induction of reactive oxygen species (ROS) by TGF-β1. IMR-90 fibroblasts were treated with TGF-β1 (200 pM, 1 h), with or without NAC or catalase pre-treatment, followed by incubation with 2′,7′-dichlorodihydrofluorescein diacetate (DCF, 10 μM, 15 min). Representative images of DCF fluorescence were shown, and ROS production was quantified and presented relative to that without any treatment as means ± SEM (n = 3; *, *P* < 0.05). Bar size, 100 μm. **B-E.** Western analyses of the effect of antioxidant pre-treatment on basal and TGF-β1-induced expression of α-SMA (B), Notch3 (C), phospho-p38 (D), and phospho-JNK1/2 (E). Cells were pretreated with NAC (4 mM, 1 h) or catalase (1000 U/L, 4 h) prior to TGF-β1 (200 pM) stimulation for 48 h (for α-SMA and Notch3) or 1 h (for p38 and JNK1/2). The expression levels were quantified and normalized with their respective controls, and presented relative to that treated with TGF-β1 alone as means ± SEM (n = 3-4; *, *P* < 0.05). **F-G.** Expression of phosphorylated ERK1/2, p38, and JNK1/2 in IMR-90 fibroblasts treated with NAC (F, 4 mM) or catalase (G, 1000 U/L) for 0-240 min.

Consistent with a previous report showing induction of ROS by TGF-β1 [14], we found that levels of ROS in IMR-90 fibroblasts were increased upon TGF-β1 stimulation (Figure 3A). Pre-treatment with NAC or catalase suppressed TGF-β1-induced ROS formation (Figure 3A), α-SMA expression (Figure 3B), Notch3 expression (Figure 3C), and phosphorylation of p38 and JNK1/2 (Figure 3D and 3E). Therefore, TGF-β1 induces IMR-90 fibroblast differentiation through p38, JNK1/2 and Notch3 in a manner depending on ROS.

### ERK1/2 signaling suppresses ROS-dependent basal differentiation of IMR-90 fibroblasts

Because α-SMA was intrinsically expressed and could be suppressed by NAC or catalase in the absence of TGF-β1 treatment (Figures 1A and 3B), we next determined the impact of ROS and MAPK/Notch3 signaling on basal IMR-90 fibroblast differentiation. Interestingly, we observed a burst of ERK1/2 activation upon NAC or catalase treatment (Figures 3F and 3G), indicating that ERK1/2 signaling was suppressed by basal ROS in IMR-90 fibroblasts. In stark contrast, basal levels of phosphorylated p38 and JNK1/2 were not affected by NAC or catalase (Figures 3F and 3G). Basal Notch3 level was suppressed by NAC, but not catalase (Figure 3C).

**Figure 4:**
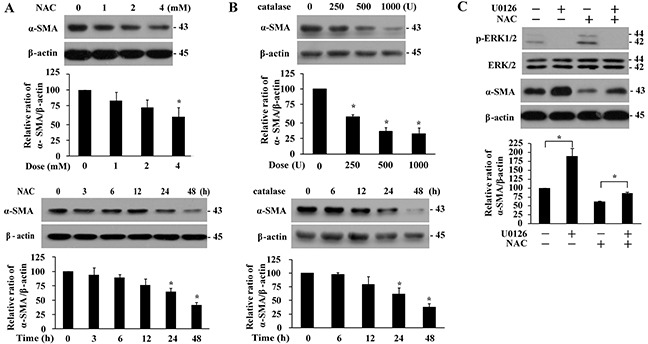
Basal α-SMA expression is suppressed in the presence of antioxidant or by ERK signaling Dose- and time-dependent effect of NAC **(A**, 48 h, upper panels; 4 mM, lower panels) and catalase (**B**, 48 h, upper panels; 1000 U/ml, lower panels) treatment on α-SMA protein levels in IMR-90 cells. **C.** Effect of U0126 (10 μM, 1 h) on ERK1/2 phosphorylation and α-SMA protein levels in the presence or absence of NAC (4 mM, 48 h) pre-treatment. The expression levels were quantified and normalized with their respective controls, and presented relative to that without any treatment as means ± SEM (n = 3-4; *, *P* < 0.05 compared to controls).

Treatment of IMR-90 fibroblasts with NAC or catalase resulted in time- and dose-dependent decreases in α-SMA protein levels (Figures 4A and 4B), suggesting that basal ROS was required for differentiation into myofibroblasts. Subsequent analyses demonstrated that inhibition of ERK1/2 signaling by U0126 elevated α-SMA protein levels in the presence or absence of NAC (Figure 4C), indicating an inhibitory role of ERK1/2 in basal fibroblast differentiation. Taken together, ERK1/2 signaling, but not p38/JNK-Notch3 cascade, limits differentiation of IMR-90 fibroblasts at basal levels in a ROS-dependent manner.

### ERK1/2 modestly inhibits TGF-β1-induced fibroblast differentiation and collagen gel contraction

**Figure 5:**
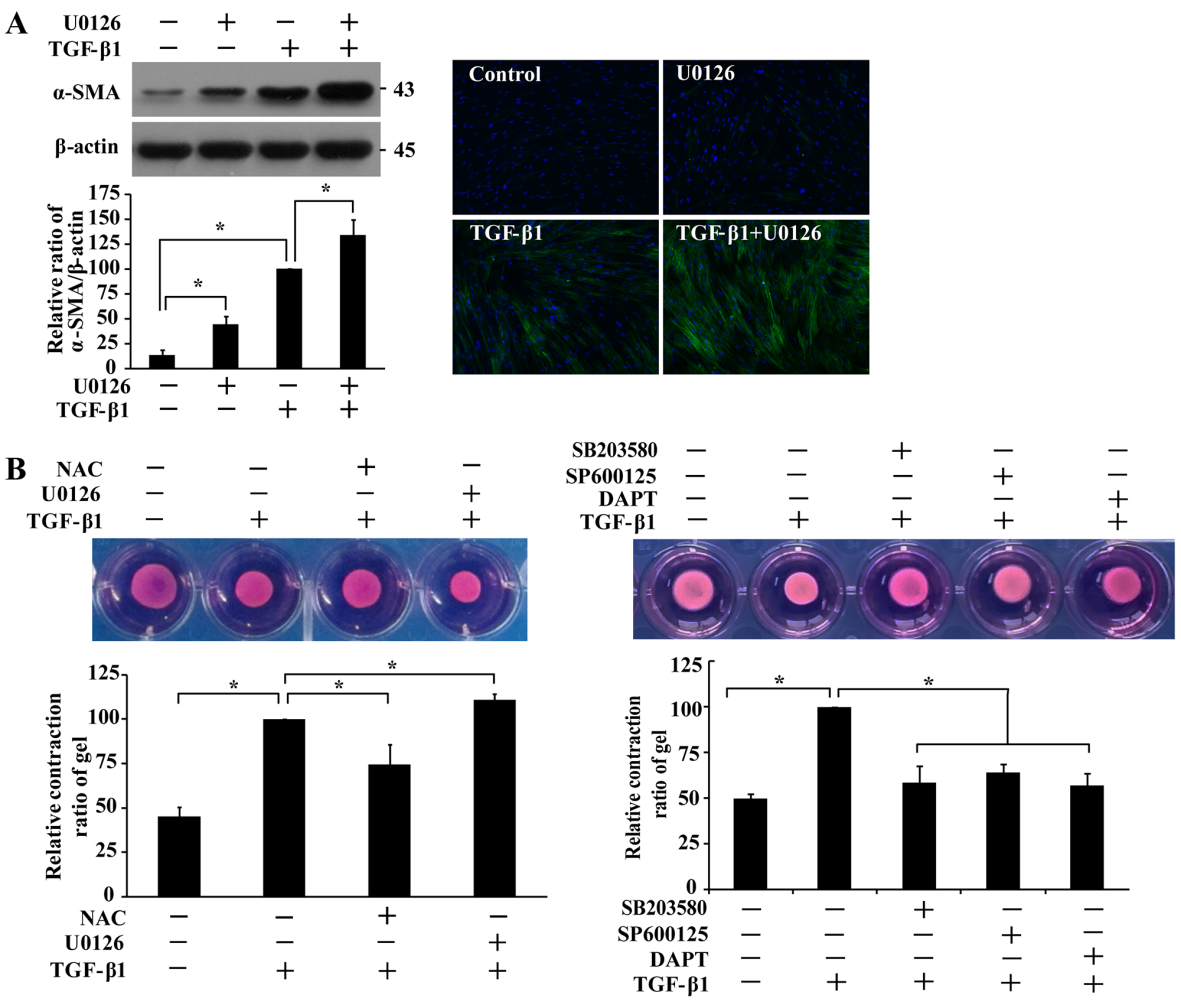
ERK1/2 inhibits TGF-β1-induced α-SMA expression and collagen gel contraction **A.** IMR-90 cells were pre-treated with U0126 (10 μM, 1 h) prior to TGF-β1 treatment (200 pM, 48 h), followed by Western and immunofluorescence analyses. α-SMA bands were quantified and normalized with those of β-actin, and presented relative to that with only TGF-β1 treatment as means ± SEM (n = 3-4; *, *P* < 0.05). **B.** IMR-90 cells were cast into three-dimensional collagen gels and floated in medium containing NAC (4 mM), U0126 (10 μM), SB203580 (10 μM), SP600125 (20 μM) or DAPT (10 μM) for 1 h prior to treatment with TGF-β1 (200 pM). Gel size was measured daily and representative photographs on day 3 of collagen gel contraction assay were presented. Relative contraction was calculated based on the formula, [(area at day 0 − area at day 3)/area at day 0] × 100%, and normalized to that treated with TGF-β1 only. Values are means ± SEM (n = 4). *, *P* < 0.05.

While ERK1/2 was not activated by TGF-β1 (Figure 1C), there was an inhibitory role of ERK1/2 in basal a-SMA expression (Figure 4C). To elaborate on these observations, we next studied the effect of ERK1/2 on TGF-β1-induced differentiation. Pre-treatment of IMR-90 fibroblasts with an ERK1/2 inhibitor U0126 increased a-SMA protein levels in the presence or absence of TGF-β1 treatment as demonstrated by Western and immunofluorescent analyses (Figure 5A). The pathogenesis of lung fibrosis is closely related to the contraction characteristics of lung fibroblasts, which can reduce pulmonary compliance and limit stretch and expansion of the lung. Increased α-SMA expression is known to enhance fibroblast contractile activity [15]. Results from three-dimensional collagen gel contraction assays demonstrated that TGF-β1-induced contraction of IMR-90 fibroblasts was inhibited by NAC, SB203580, SP60012, or DAPT but modestly yet significantly (*P* < 0.05) augmented by U0126 pre-treatment (Figure 5B). Thus, ERK1/2 inhibits TGF-β1-induced differentiation of fibroblasts, which is opposite of p38, JNK1/2 and Notch3.

### Differential impacts on basal and TGF-β1-induced expression of extracellular matrix proteins by MAPKs or Notch3 in IMR-90 cells

**Figure 6:**
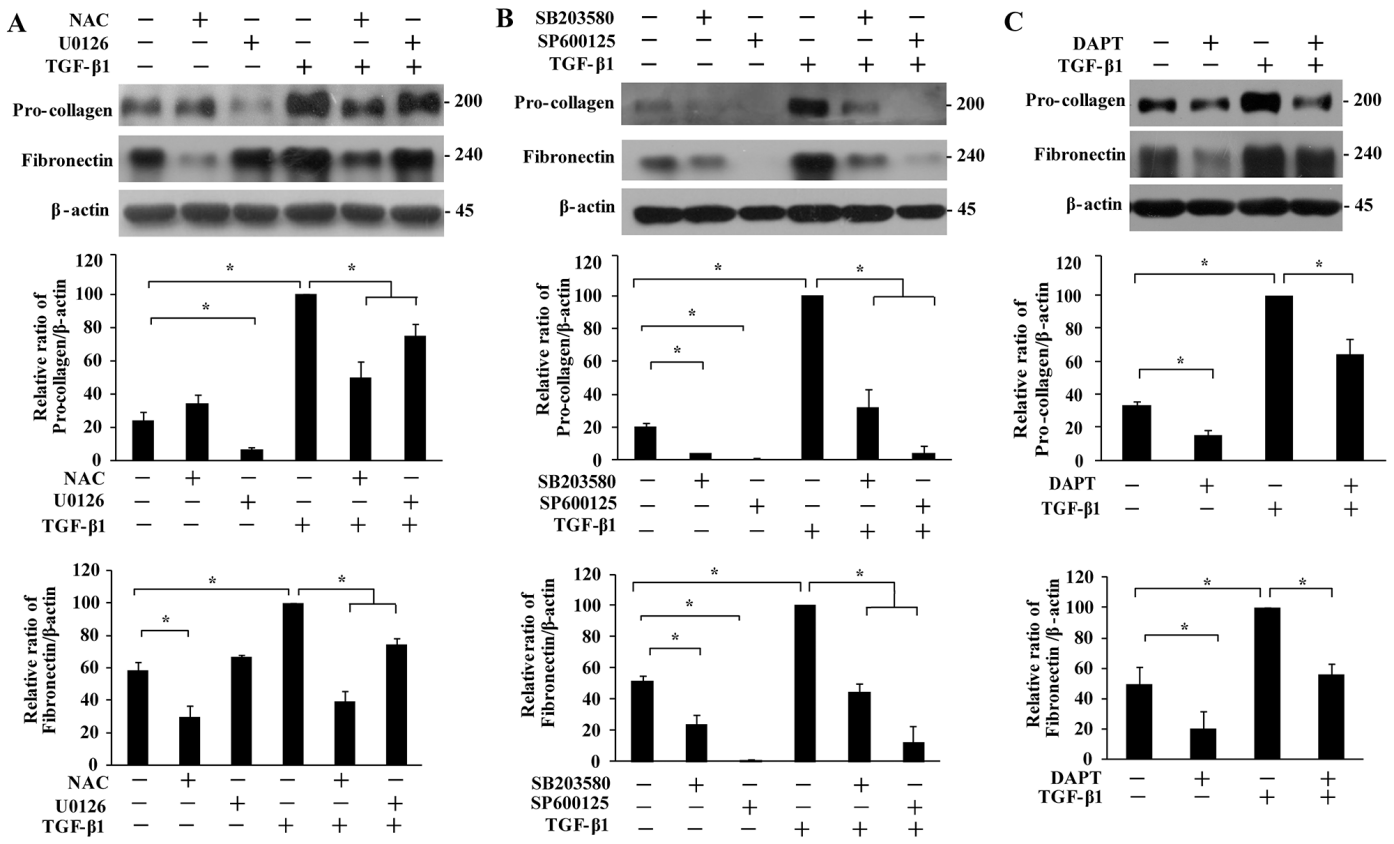
Effect of MAPKs and Notch3 on TGF-β1-induced secretion of extracellular matrix proteins IMR-90 cells were pre-treated with NAC (**A**, 4 mM), U0126 (A, 10 μM), SB203580 (**B**, 10 μM), SP600125 (B, 20 μM) or DAPT (**C**, 10 μM) for 1 h, followed by TGF-β1 treatment (200 pM, 48 h). The pro-collagen and fibronectin bands were analyzed by Western analysis, quantified, normalized to those of β-actin, and presented relative to that of TGF-β1 treatment only as means ± SEM (n = 3). *, *P* < 0.05.

To further characterize pulmonary fibrosis and confirm the roles of different MAPKs and Notch3 in the pathogenesis, markers of extracellular matrix were assessed. Treatment of IMR-90 fibroblasts with TGF-β1 resulted in elevated abundance of pro-collagen and fibronectin in the cells. Such induction of extracellular matrixes was greatly reduced when the cells were pre-treated with SP600125 or SB203580 (Figure 6B), but only modestly with U0126 (Figure 6A) or DAPT (Figure 6C). In the absence of TGF-β1 treatment, basal pro-collagen levels were reduced by any of the MAPK and Notch inhibitors whereas levels of fibronectin were reduced by pre-treatment with SP600125, SB203580 or DAPT but not U0126. In the presence of NAC, levels of pro-collagen and fibronectin were reduced after TGF-β1 treatment, but only fibronectin was down-regulated in the absence of TGF-β1 (Figure 6A).

### Increased expression of TGF-β1, α-SMA, p-MAPKs, and Notch3 in pulmonary fibroblasts from patients with IPF and bleomycin-injured mice

**Figure 7:**
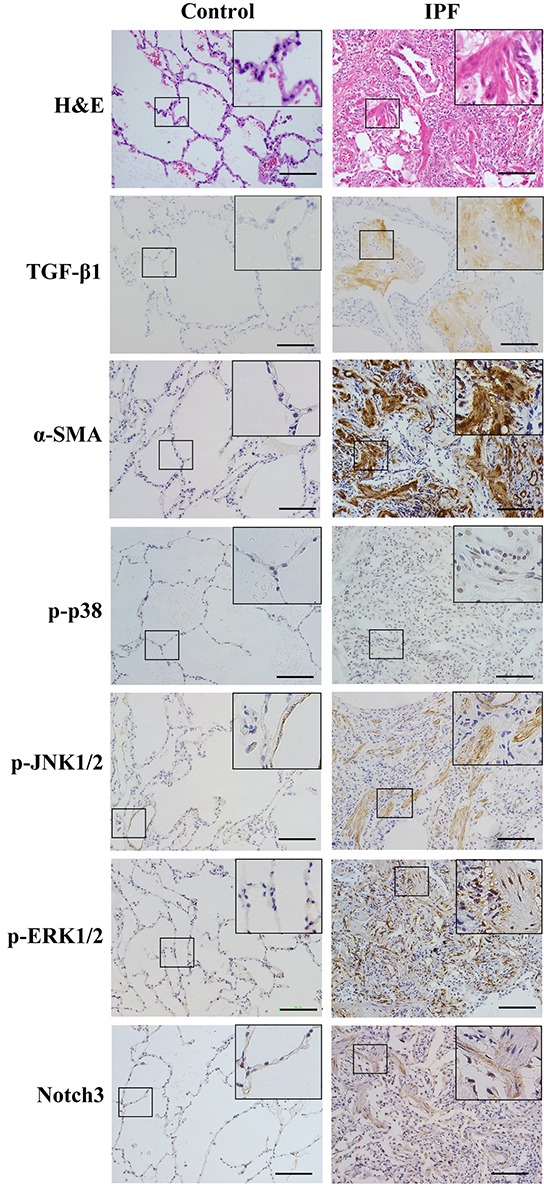
Representative images of immunohistochemical analyses of p38, JNK1/2, and ERK1/2 phosphorylation, α-SMA, TGF-β1 and Notch3 proteins and H&E staining in paraffin-embedded lung sections from patients with idiopathic pulmonary fibrosis (IPF) and normal lung parenchyma Bar szie, 100 μm.

To verify clinical relevance of our findings from cultured cells, we determined TGF-β1, α-SMA, phosphorylation status of p38, JNK1/2 and ERK1/2, and Notch3 expression in IPF and normal lungs (Figure 7). Compare to sections from normal individuals, results from H&E staining showed architectural destruction and dense fibrosis in the lungs of IPF patients. TGF-β1 was detectable in epithelial cells, where there was evidence of extensive interstitial inflammation and fibrosis. While p-p38 was detected predominantly in the nuclei of fibroblasts or myofibroblasts and epithelial cells, p-JNK1/2 was observed in the cytoplasm of fibroblasts or myofibroblasts and the surrounding blood vessels. p-ERK1/2 immunoreactivity was localized in the cytoplasm of fibroblasts or myofibroblasts and vascular endothelial cells. Notch3 was found in the cytoplasm, membrane and nuclei of fibroblasts or myofibroblasts and epithelial cells. Although healthy lungs also expressed p-JNK1/2, Notch3 and α-SMA in the vicinity of blood vessels as shown in IPF lungs, TGF-β1, p-p38 or p-ERK1/2 were not detected in healthy lungs.

**Figure 8:**
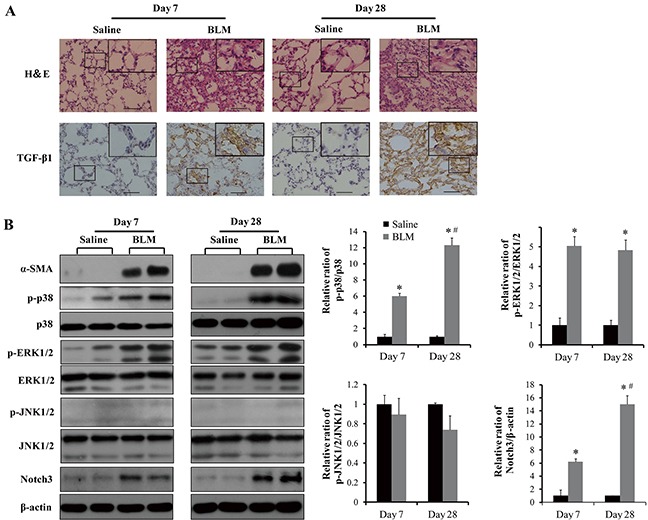
Analyses of phosphorylation of MAPKs, TGF-β1 and Notch3 expression in lungs of (BLM) bleomycin-injured and control mice **A.** Representative images of H&E staining and TGF-β1 expression determined by immunohistochemistry. Bar size, 100 μm. **B.** Western blot analyses of p38, JNK1/2 and ERK1/2 phosphorylation, α-SMA and Notch3 expression. The protein bands were quantified, normalized with their non-phosphorylated counterparts or β-actin, and presented relative to that of saline-treated controls as means ± SEM (n = 3-4). *, *P* < 0.05 vs saline. #, *P* < 0.05 vs day 7.

Bleomycin is often used to induce pulmonary fibrosis although this model may not be authentic for IPF [16]. We thus determined the above proteins in the lungs of mice intratracheally injured by bleomycin to further study the activation status of these signaling events (Figure 8). Compare to the control sections, results from H&E staining showed architectural destruction and dense fibrosis in the lungs at both day 7 and 28 after bleomycin treatment. However, TGF-β1 showed a different pattern of distribution in bleomycin-induced pulmonary fibrosis compared with that in IPF patients (Figures 7 and 8A). The levels of α-SMA, p-p38, p-ERK1/2, and Notch3, but not p-JNK1/2, in the lungs were obviously induced by bleomycin treatment at both day 7 and 28 (Figure 8B, left panel). In the bleomycin-injured lungs, levels of p-p38 and Notch3, but not p-ERK1/2, were significantly increased (*P* < 0.05) at day 28 than at day 7 (Figure 8B, right panel).

## DISCUSSION

One of the major histological patterns of usual interstitial pneumonia (UIP) is IPF, which is exemplified by the formation and persistent presence of myofibroblasts. Therapies with glucocorticoids and other immunomodulatory agents are largely ineffective for this disease [12]. It is thus thought that IPF is not significantly attributed to inflammatory responses; rather, other intracellular pathways and mediators may play a key role in the pathogenesis of UIP. While p38 and JNK are previously known to contribute to fibroblast differentiation, results from the current study present two novel findings: 1) the promotion of myofibroblast formation by a ROS-dependent and MAPK-regulated Notch3 signaling; 2) a dual role of ERK1/2 during lung fibrosis that inhibits fibroblast differentiation but promotes extracellular matrix formation.

The signaling cascades of MAPK are often involved in TGF-β1 signaling and the associated cellular processes [17,18]. Our results are consistent with previous reports showing JNK and p38 activation upon TGF-β1-stimulated myofibroblast differentiation [19–22]. Strikingly, although ERK1/2 has been shown to be activated upon TGF-β1 stimulation in other cells [18,23], here we demonstrate that ERK1/2 activation predominantly inhibits basal differentiation of IMR-90 cells in a ROS-dependent manner. Notch signaling has been linked to fibrotic diseases [11,24,25], examples of which include Notch1 promotion of differentiation in murine lung fibroblasts [24] and induction of epithelial–mesenchymal transition with increased migratory behavior in pulmonary fibrosis [11]. Interestingly, we show that Notch3, rather Notch1, is up-regulated in human IMR-90 fibroblasts upon TGF-β1 stimulation and in IPF patients with an elevated expression of TGF-β1. Notch3 is known to be involved in liver and kidney fibrosis [26,27], suggesting an important role of Notch3 in regulating fibrotic formation. While Notch3 is regulated by p38 in vascular smooth muscle cells [28], TGF-β1-induced Notch3 up-regulation in fibroblasts is also downstream of JNK signaling. However, fibroblast differentiation and expression of extracellular matrix protein by Notch3 stimulation are less significant compared to those activated by p38 and JNK, implying the MAPK-mediated fibrosis only partially involves Notch3 signaling. Interestingly, the extent of gel contraction inhibition by Notch3 is similar to those by p38 and JNK, which may attribute to the regulation of Notch3 in mechanosensitivity [28].

Another intriguing observation is the doublet of Notch3 proteins at 83 and 90 kDa by using an antibody against its C-terminus. While the 90 kDa Notch3 represents the intracellular monomer after S1 cleavage by furin, the nature of the 83 kDa Notch3 remains puzzling. To understand whether the 83 kDa Notch3 is the intracellular active domain after S3 cleavage by γ-secretase, we pretreated the cells with γ-secretase inhibitor DAPT but observed no shifting of these bands (data not shown). Also, based on the size (90 amino acids or 9.7 kDa) between the S1 and S3 cleavage sites, the predicted molecular weight of the intracellular active domain is 80.3 kDa. Thus, the 83 kDa Notch3 band seems to be attributed to post-translational modifications or a yet-to-be-identified proteolytic product. Although it is of future interests to elucidate the nature of these Notch3 bands observed herein in IMR-90 fibroblasts, similar patterns of Notch3 proteins have been reported in HEK293, H460 and HeLa cells [29].

TGF-β-induced profibrotic responses are critically mediated by ROS [30–32]. Results from our current study demonstrate the involvement of ROS in TGF-β1-induced fibroblast differentiation and TGF-β1-induced activation of p38 and JNK and Notch3 signaling, consistent with those reported previously by using endothelial [31] and vascular smooth muscle cells [28]. A key observation reported herein is the critical role of ERK1/2 activation in the limitation of basal fibroblast differentiation under decreased oxidative stress. Such ERK1/2 function is in stark contrast to that of p38 and JNK, as they are mainly activated after TGF-β1 induction and increased oxidative stress but not by changes in basal ROS status. Indeed, in other cell types such as primary cortical neurons and embryonic PC12 cells, NAC treatment is associated with increased expression of phosphorylated MAPK [33,34]. Consistent with a previous study showing inhibition of cytokine-induced activation of JNK and p38 but activation of ERK1/2 pathway by NAC [35], our data suggest that low ROS status, either at a basal level or even lower after antioxidant treatment, is in favor of ERK1/2 activation (Figures 3F and 3G), which, in turn, can suppress spontaneous fibrosis. Interestingly, it seems incomprehensive that ROS status at basal level is inversely associated ERK1/2 activation because TGF-β1 stimulates ROS production but ERK1/2 activation remains unchanged. One plausible explanation is that TGF-β1 activates ERK1/2 via a ROS-independent pathway that offsets ROS-induced ERK1/2 inhibition by TGF-β1. As a consequence, ERK1/2 activation remains unchanged after TGF-β1 treatment. Of note, NAC is used for adjunct therapy to ameliorate lung fibrosis when administrated together with prednisolone and azathioprine [36]. In addition to the well-known antioxidative role of NAC, our results suggest that the pharmacological function of NAC may act through the activation of ERK1/2 signaling to inhibit fibroblast differentiation. We also show that ERK regulates Notch3 signaling independent of TGF-β1, suggesting an intriguing and important functional role of ERK in fibroblast differentiation. It is noteworthy that NAC per se decreases the level of Notch3 independent of ROS [37]. However, such decline seems not to affect TGF-β1-induced MAPK activation based on the results of DAPT pre-treatments.

Such antagonistic role of ERK1/2 in pulmonary differentiation is consistent with previous studies in other cell types [38,39]. However, the inhibitory role of ERK1/2 in differentiation does not occur universally, as MEK inhibition by PD98059 has been shown to reduce lung injury and inflammation in a mouse model of pulmonary fibrosis induced by bleomycin [40]. Furthermore, in the TGF-α-induced lung fibrosis model, ERK1/2 inhibition prevents the progression of established fibrosis [41]. To reconcile this seemingly inconsistency, we speculate that the initial secretion of pro-inflammatory cytokines and mediators following early fibrotic triggers such as epithelial-mesenchymal transition would activate ERK1/2, contributing to lung injury and inflammation. However, at later stages in the pathogenesis of IPF when fibroblast differentiation dominates, inhibition of ERK1/2 likely potentiates the progression of IPF. Indeed, unlike p38 and Notch3 showing persistent activation from day 7 to 28 in the bleomycin-injured mice, ERK1/2 signaling remains unaffected, if not inhibited, at these two time points. Although being considered by many as the fittest murine model of IPF [42], this bleomycin-induced pre-clinical model may not be authentic for IPF but serves as one for patients with active, instead of progressive, fibrosis [16]. Patterns of TGF-β1 distribution in lungs of bleomycin-injured mice and IPF patients differ, since fibrosis patients are usually diagnosed only when the disease has progressed to a more advanced stage with interstitial inflammation and tissue regeneration [43]. Also, as being previously noted [44], a non-significant increase of phosphorylated JNK1/2 shows in this animal model, probably due to a differential involvement of JNK pathway in inflammatory lung injuries [44]. Furthermore, all potential anti-fibrotic compounds should be evaluated in the phase of established fibrosis rather than in the early period of bleomycin-induced inflammation for assessment of its antifibrotic properties [45]. While we are limited by the lung tissue samples of IPF patients to temporally analyze the changes of ERK1/2 activation, results from a previous study indeed suggest that ERK1/2 activation in epithelial and endothelial cells subsides accompanying the progression of fibrosis in IPF lungs [46].

**Figure 9:**
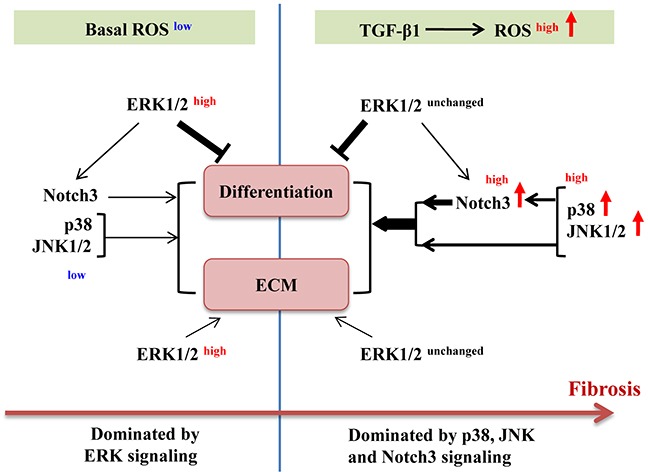
Schematic illustration of roles of ERK1/2, p38, JNK1/2 and Notch3 in basal and TGF-β1-induced myofibroblast differentiation ECM, extracellular matrix; ROS, reactive oxygen species; →, lead to/activate; 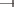, inhibit. Line thickness indicates the extent of impact. Superscripts indicate the level or activation status.

Taken together, our results suggest a sophisticated participation of different MAPKs and Notch3 in maintaining and regulating fibroblast differentiation during fibrosis. As depicted in Figure 9, a basal, low ROS level sets ERK1/2 in motion, which subsequently inhibits fibroblast differentiation through dominance over p38, JNK and Notch3 signals favoring differentiation. Under the circumstance of TGF-β1 stimulation, the ROS level is dramatically elevated, resulting in enhancement of p38 and JNK1/2 activation and Notch3 expression. Consequently, the stimulatory signals including p38, JNK1/2 and Notch3 take over ERK1/2 to dictate cells towards fibrosis. To the best of our knowledge, the above evidence-based scheme is for the first time proposed toward understanding the pathogenesis of IPF, as well as to provide therapeutic potential of antioxidant and MAPK/Notch3 inhibitors in combination.

## MATERIALS AND METHODS

### Cells and chemicals

Human IMR-90 lung fibroblasts were purchased from American Type Culture Collection (Manassas, VA, USA) and cultured in DMEM (Invitrogen, Carlsbad, CA, USA) supplemented with 10% fetal bovine serum (Invitrogen), penicillin (100 U/ml), and streptomycin (100 μg/ml) (Solarbio Science and Technology, Beijing, China) at 37°C in a humidified incubator with 5% CO_2_. Cells at passages 7-13 were starved overnight in serum free medium before treatments. We purchased recombinant human TGF-β1 peptide from Invitrogen (Carlsbad, CA, USA), *N*-acetyl-_L_-cysteine (NAC) and SP600125 from Beyotime (Shanghai, China), bovine liver catalase from Sigma (St. Louis, MO, USA), N-[N-(3,5-difluorophenacetyl)-_L_-alanyl]-S-phenylglycin *t*-butyl ester (DAPT) from Calbiochem (San Diego, CA, USA), SB203580 from Merck (San Diego, CA, USA), U0126 from Cell Signaling (Boston, MA, USA) and bleomycin from Selleck Chemicals (Houston, TX, USA). Cells were pretreated with NAC (4 mM), SP600125 (20 μM), SB203580 (10 μM), U0126 (10 μM), or DAPT (10 μM) for 1 h, or catalase (1000 U/ml) for 4 h prior to TGF-β1 (200 pM) stimulation.

### ROS measurement

To assess intracellular ROS production, cells were incubated with 2′,7′-dichlorodihydrofluorescein diacetate (DCF, 10 μM, Beyotime) at 37°C for 20 min in serum free medium following treatments. Cells were then washed three times with PBS and immediately imaged under a fluorescence microscope with NIS-Elements D Software. In separate experiments, cells were collected following the PBS wash, transferred to a 96-well black-walled plate, and immediately applied to Fluoroskan Ascent FL Microplate Reader (Thermo Scientific, Waltham, MA) with excitation at 485 nm and emission at 538 nm.

### Transfection

Cells were transfected with siRNA sequences using Lipofectamine 2000 (Invitrogen, Carlsbad, CA, USA) according to the manufacturer's protocol. Scrambled and Notch3 siRNAs (5′-CCUGGCUACAA UGGUGAUATT-3′) were purchased from GenePharma (Shanghai, China). After transfection, cells were cultured for 24 hours in complete medium prior to overnight starvation and then drug treatment.

### RNA isolation and mRNA analyses

Total RNA was extracted using TRIzol reagent (Invitrogen) and reverse-transcribed to cDNA using a kit from Tiangen (Tianjin, China). HES1 and HRT1 mRNAs were amplified with the following primer pairs: HES1, 5′-GTCAACACGACACCGGATAA-3′ and 5′-GAGGTGCTTCACTGTCATTTCC-3′; HRT1, 5′-TGACCGTGGATCACCTGAAA-3′ and 5′-GCTGGG AAGCGTAGTTGTTG-3′; β-actin, 5′-GTGGGGCGC CCCAGGCACCA-3′ and 5′-CTTCCTTAATGTCACGCACGATTTC-3′. PCR was performed at an initial denaturation temperature at 94°C for 3 min, then 35 cycles at 94°C for 30 sec, 48°C (for HES1 and HRT1) or 58°C (for β-actin) for 30 sec, and 72°C for 30 sec, and a final extension at 72°C for 5 min. The PCR products were separated by a 2% agarose gel containing ethidium bromide and visualized under a gel imaging system.

### Western blot

Cells were lysed in a sample buffer containing SDS (2%) and Tris-HCl (60 mM, pH 6.8). Lysates were boiled for 5 min and total protein concentration was measured by using a BCA kit (Beyotime). Western analysis was performed as previously described [28] by using antibodies against Notch3 (sc-5593), α-SMA and pro-collagen (Santa Cruz Biotechnology, Santa Cruz, CA, USA), fibronectin (Sigma), and p-JNK1/2, p-p38, p-ERK1/2, JNK1/2, p38, ERK1/2, and β-actin (Cell signaling), and a chemiluminescence detection kit (LumiGLO® Reagent and Peroxide, Cell signaling).

### Immunofluorescence

Cells were fixed in cold paraformaldehyde (4%) for 15 min, followed by permeabilization in Triton X-100 (0.5%) for 20 min. After blocking with 5% bovine serum albumin (Beyotime) for 1 h at 4°C, cells were sequentially incubated with anti-α-SMA antibody (1:200 dilution) overnight at 4^°^C and Alexa Fluor® 488-conjugated goat anti-mouse IgG antibody (Invitrogen) for 1 h at 4°C. Cells were then counterstained with Hoechst 33258 (1:2000 dilution; Beyotime) to locate the nuclei and mounted with anti-fade mounting media (Beyotime).

### Bleomycin-injured mouse model of lung fibrosis

Male C57BL/6 mice (8-9 weeks of age, 20-23 g) were housed three to four per cage and given free access to pelleted chow diet and distilled water in an animal room maintained at constant temperature and humidity with a 12-h light-dark cycle. All animal experiments were approved by the Institutional Laboratory Animal Care and Use Committee at Wenzhou Medical University. A bleomycin-induced mouse model of lung fibrosis was generated following the protocol as described previously [47]. In brief, mice were anesthetized using 2.5% chloral hydrate and then intratracheally administered with 50 μl of bleomycin (5 mg/kg body weight) dissolved in sterile saline or only sterile saline in control mice. Mice were sacrificed at 7 or 28 days following the administration. Lungs were collected, and then fixed or homogenized in the sample buffer.

### Immunohistochemistry

Histological sections were obtained from paraffin-embedded lung biopsies of two controls and two IPF patients with institutional review board approval and informed written consent. Clinical criteria to identify IPF were based on the identification of a typical interstitial pneumonia pattern. Due to ethical issues, normal lung tissues from patients with lung cancer, instead of healthy individuals, were obtained and considered as the controls. In the bleomycin-induced model, mice were euthanized and perfused via the right ventricle with 10 ml of saline. Lungs were removed, fixed overnight in formalin before being dehydrated in ethanol, and processed using standard procedures for paraffin-embedded samples. The antibody against TGF-β1 was from Santa Cruz Biotechnology, and others were the same as described above. Immunohistochemistry was performed by using a Histostain-SP Kit (Zymed Laboratories Inc., San Francisco, CA, USA) according to the manufacturer's instructions.

### Collagen gel contraction assay

Collagen gel was reconstituted using rat tail collagen type I (BD Biosciences, Bedford, MA) and cell suspension prepared in complete medium. Aliquots of the mixture (0.5 ml/well) were then distributed to a 24-well plate and allowed to polymerize for 30 min at 37°C before adding serum-free medium (1 ml) to each of the wells. Following overnight starvation, the cells were pretreated as indicated and then stimulated with TGF-β1 for 3 days. Data were expressed as the relative contraction ratio normalized with the area treated with TGF-β1 only.

### Statistical analysis

Data were analyzed by using Predictive Analytics Software 18.0 (PASW, version 18.0) for Windows. A one-way analysis of variance with least significant difference test was applied to determine statistical significance (*P* < 0.05).

## Abbreviations

α-SMA: α-smooth muscle actin
DCF: 2′,7′-dichlorodihydrofluorescein diacetate
IPF: idiopathic pulmonary fibrosis
MAPK: mitogen-activated protein kinase
NAC: *N*-acetyl-_L_-cysteine
ROS: reactive oxygen species
TGF-β1: transforming growth factor β1
UIP: usual interstitial pneumonia
